# In Memoriam: George Martin Baer (1936–2009)

**DOI:** 10.3201/eid1508.090897

**Published:** 2009-08

**Authors:** Brian W.J. Mahy

**Affiliations:** Centers for Disease Control and Prevention, Atlanta, Georgia, USA

**Keywords:** commentary, rabies, obituary, George Martin Baer

George Martin Baer, DVM, MPH, who for many years was chief of the Rabies Laboratory in the Division of Viral and Rickettsial Diseases at the Centers for Disease Control and Prevention (CDC), Atlanta, Georgia, USA, died suddenly at his home in Mexico City on June 2, 2009, at the age of 73. He was an eminent veterinary virologist whose book, The Natural History of Rabies, the second edition of which was published in 1991, is the major reference work in the field.

He has been widely acclaimed as “the father of oral rabies vaccination” as a result of his discovery of a strain of rabies virus (SAD strain) that immunizes foxes when given into the mouth cavity. His intensive work on the development of baits for vaccine administration to canines paved the way for successful mass fox vaccination campaigns conducted in Switzerland, several other European countries, and Canada.

Dr Baer was born in London in 1936, but after the outbreak of World War II his family moved to the United States, and he was brought up in New York. He attended Cornell University in Ithaca, New York, and received a bachelor of science degree in agricultural sciences in 1954 and a Doctor of Veterinary Medicine degree in 1959. In 1961, he earned a Master of Public Health degree from the University of Michigan at Ann Arbor. He then came to CDC as an Epidemic Intelligence Service Officer, initially assigned to the New York State Health Department in Albany, where he first became interested in rabies. He subsequently joined the CDC Southwest Rabies Investigations Laboratory in Las Cruces, New Mexico, and from 1966 through 1969 was a consultant for the Pan American Health Organization in Mexico. He returned to CDC in Atlanta in 1969 to become chief of the Rabies Laboratory and served until his retirement in August 1991. He and his family then moved to Mexico City, where he founded a diagnostic laboratory and continued to use his expertise on rabies as a member of the Mexican International Steering Committee for the Rabies in the Americas Conference.

Dr Baer was a valued supporter of and reviewer for Emerging Infectious Diseases and will be greatly missed. We offer our condolences to his wife, Maria Olga, and daughters, Katherine, Alexandra, and Isabella, as well as to his 4 granddaughters.

